# An unusual cause of shoulder pain in an elderly woman: a case report

**DOI:** 10.1186/1752-1947-7-271

**Published:** 2013-12-13

**Authors:** Nessrine Akasbi, Mohammed Elidrissi, Latifa Tahiri, Abdelmajid Elmrini, Taoufik Harzy

**Affiliations:** 1 Rheumatology Department, Hassan II University Hospital, Fez 30070, Morocco; 2 Orthopedic Surgery Department, Hassan II University Hospital, Fez Morocco

**Keywords:** Clavicle, Etiology, Insufficiency fracture, Stress fracture

## Abstract

**Introduction:**

Stress fracture of the clavicle is a rare entity. It can manifest itself with an atypical shoulder pain. The clavicle is vulnerable to pathological fractures from several causes such as neoplasm, infection and rarely metabolic bone disease.

**Case presentation:**

We report a case of a bone insufficiency fracture of the clavicle, in a 67-year-old Moroccan woman patient with several risk factors of bone insufficiency including osteoporosis, osteomalacia and primary hyperparathyroidism.

**Conclusion:**

The diagnosis of bone insufficiency-related fracture of the clavicle can be challenging. It should be considered in the differential diagnosis of shoulder or clavicle pain.

## Introduction

Stress fractures are common lesions that occur without high energy trauma or focal abnormality. They include fatigue fractures which are caused by the application of abnormal stress to a bone with normal resistance, and insufficiency fractures which occur when normal activity stresses a bone that is deficient in resistance [1].

Insufficiency fractures of the clavicle are particularly rare. Only a few cases have been reported. Also, medial clavicle fractures are uncommon and are normally caused by high-energy trauma. A low impact mechanism of injury should lead one to suspect a pathological fracture [2]. Many reports about insufficiency fractures of the clavicle indicate several predisposing activities in which the clavicle is subject to repetitive shearing forces, especially in sporting activities [3].

We report the case of a patient with a stress insufficiency fracture of the clavicle, in which the diagnosis was delayed because of the pain’s localization to the shoulder and multiple predisposing risk factors including osteoporosis, osteomalacia and primary hyperparathyroidism.

## Case presentation

**Figure 1:**
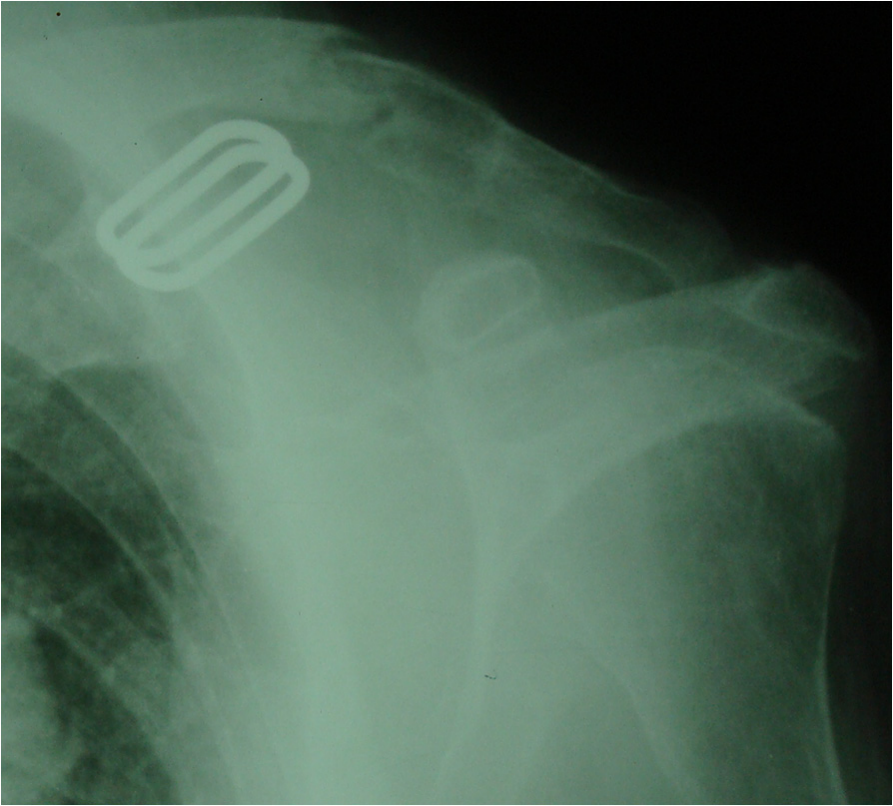
Medial diaphyseal heterogenous bone defect with a fracture.

A self sufficient 67-year-old postmenopausal, Moroccan woman, with no past medical history of carcinoma, presented to our emergency department with severe mechanical right-sided pain affecting her shoulder with functional limitation. Our patient had a history of primary hyperparathyroidism for the last 15 years, diagnosed on the clinical, laboratory and radiological findings. She also had osteoporosis (T-score at the hip; -3, 3 and T-score at the wrist: -3, 2), but she was not receiving any antiosteoporotic treatment. The patient's shoulder pain began two weeks previously, without any trauma, repetitive stress or trigger factors, and worsened with time. On physical examination her clavicle was tender on palpation, without any swelling or inflammatory signs. She had no systemic symptoms and the rest of the physical examination was normal. A shoulder X-ray showed a medial diaphyseal heterogenous and slightly osteodense bone defect of her right clavicle, with a fracture at the level of the lesion (Figure 1). This was considered malignant and urgently investigated further. An X-ray of her entire skeleton did not find any fracture or abnormalities. A complete blood count was normal, with no evidence of an inflammatory syndrome: erythrocyte sedimentation rate and level of C-reactive protein were normal, there was no abnormal peak on serum proteins electrophoresis, and her liver and kidney function tests were normal. Tests for tumoral markers were negative, a urine analysis for Bence-Jones proteins was negative, and a bone marrow aspiration to exclude lymphoma or myeloma was also normal. However, the levels of bone metabolic markers were disturbed. Her calcium level was high at 108mg/L (normal range: 95mg/L to 105mg/L), the phosphate level was 30mg/L (normal range 25mg/L to 45mg/L), there was an increase in total alkaline phosphatase at 300IU/L (normal range 30IU/L to 100IU/L), urine calcium was 40mg/24 hours (100 to 300mg/24 hours), her serum parathyroid hormone level was 438pg/mL (normal <70pg/mL), and her plasmatic 25- hydroxyvitamin D2D3 was below 6ng/mL (normal range 30 to 50ng/mL). Further diagnostic studies, including a computed tomography scan of her thorax, abdomen and pelvis, were performed to exclude an underlying malignant process but revealed no cancer. An X-ray of her pelvis demonstrated generalized demineralization and a compatible aspect of osteomalacia. A cervical ultrasound found parathyroid hyperplasia. A microbiopsy of the lesion in her clavicle was done. The anatomopathologic examination did not reveal any cancer-related disease.

Osteoporosis and hyperparathyroidism associated with osteomalacia were considered as the potential culprits of the spontaneous clavicle’s fracture in our patient. The fracture was dressed with a figure-of-eight dressing. The patient was treated with vitamin D for the osteomalacia and zoledronic acid for the osteoporosis. Surgical treatment for parathyroid hyperplasia was indicated but our patient refused it. The patient died some time after her diagnosis of unrelated issues.

## Discussion

Stress clavicle fractures are not frequent, but more common in the sporting population [4]. Other etiologies of non traumatic clavicle fractures are skeletal metastases, lymphomatous or myelomatous proliferation and infections. Clavicular metastases comprise of 6-18% of all bone metastases, particularly from renal cell carcinoma [5]. The nonspecific pain of a clavicular stress fracture referring to the upper extremity, can misdirect the clinician to more common causes of shoulder pain, such as rotator cuff and cervical disc diseases [6].

A conventional X-ray is usually normal when symptoms first present themselves. Later, a pseudotumor appears in the clavicle which corresponds to the fracture callus, that can be misdiagnosed as a tumour or infectious processes [1]. Further imaging of the clavicle such as computed tomography scan or magnetic resonance imaging is needed to adequately define the fracture and appreciate the quality of the surrounding bone. Bone scintigraphy remains an early method for detection of this injury [7].

Medial clavicular fractures are uncommon. They are the least frequent of clavicular fractures, comprising between 2% to 10% [8,9]. They are normally caused by a high-energy trauma, and are associated with other multisystem injuries, especially in men. A low impact mechanism of injury should raise suspicion of a pathological fracture of the clavicle [10].

In most of the cases reviewed, stress fractures of the clavicle, especially a medial fracture, were due to a neoplasm or infection. Benign causes like metabolic bone disease are very rare. In our patient, there were several benign factors that may have contributed to the development of this injury, notably osteoporosis, primary hyperparathyroidism and osteomalacia. Other benign predisposing risk factors of bone insufficiency could be rheumatoid arthritis, renal failure, fibrous dysplasia and algodystrophy [11]. This present case report demonstrates the difficulty in diagnosing pathological fractures of the clavicle especially an insufficiency medial fracture, which is not routinely suspected to be pathological. We have shown that a stress fracture of the clavicle can manifest itself with unusual and atypical localization of shoulder pain. In our patient, the association of several additional risk factors contributed to weaken the clavicle, to decrease the bone resistance and to predispose it to the development of a stress fracture.

## Conclusion

Stress fractures of the clavicle must be kept in mind in the differential diagnosis of shoulder and clavicle pain. Medial fractures are a rare entity, usually caused by a high energy trauma or tumor proliferation in most cases. In our patient, it was due to many additional benign risk factors.

## Consent

Written informed consent was obtained from the patient's next of kin (her son) for publication of this manuscript and accompanying images. A copy of the written consent is available for review by the Editor-in-Chief of this journal.

## Competing interests

The authors declare that they have no competing interests.

## Authors’ contributions

NA made the diagnosis, treated the patient and wrote the case report. ME participated in the treatment of the patient. LT, AE and TH supervised the writing of this paper. All authors read and approved the final manuscript.
